# Design and Analysis of a New High Precision Decoupled XY Compact Parallel Micromanipulator

**DOI:** 10.3390/mi8030082

**Published:** 2017-03-06

**Authors:** Xigang Chen, Yangmin Li

**Affiliations:** 1 Department of Electromechanical Engineering, Faculty of Science and Technology, University of Macau, Avenida da Universidade, E11, Taipa, Macao 999078, China; mb55448@umac.mo; 2 Department of Industrial and Systems Engineering, The Hong Kong Polytechnic University, Hung Hom, Kowloon, Hong Kong 999077, China

**Keywords:** high precision, two-stage amplifier, decouple, kinematic analysis, flexure mechanism design, micro/nano parallel robots, finite-element analysis (FEA) simulation

## Abstract

With the development of nanotechnology that contains automatic control, precision machinery and precise measurement, etc., micro/nano manipulation has become a new research direction in recent years. This paper presents the design and analysis procedures of a new high precision XY decoupled compact parallel micromanipulator (DCPM) for micro scale positioning applications. The DCPM is made up of the decoupler, two-stage amplifier and the piezoelectric translator (PZT) actuators, which utilizes the characteristics of flexure hinges. In this paper, firstly, a new two-stage bridge-principle amplifier is proposed by a serial connection of two fundamental bridge amplifiers in order to increase the ratio of amplification. It is pivotal for designing the micromanipulator. Then, the kinematic modeling of the micromanipulator is carried out by resorting to stiffness and compliance analysis via matrix method. Finally, the performance of the micromanipulator is validated by finite-element analysis (FEA) which is preliminary job for fabricating the prototype and designing the control system of the XY stage that is expected to be adopted into micro/nano manipulations.

## 1. Introduction

In recent years, the research activities of micro/nano operations are increasing rapidly due to micro-nano technology extensive applications. Particularly, the micro-nano scale of manipulators with ultrahigh precision play more and more important roles in some fields such as the micro-coordinate measuring machines [1,2], the adaptive adjustment mechanism in astronomical telescope, the micro-electro-mechanical system (MEMS), biomedical operation equipment, etc. In addition, we can find more specific applications in the literature [3,4,5,6,7,8]. It is easy to observe that most of these micro/nano manipulators adopt flexure hinges to compose its mechanism structure in the above literature. The reason is that flexure hinges have some advantages compared with traditional mechanical joints. For example, the compliant mechanisms based on flexure joints have wide applications in science and engineering because of their many virtues including easily manufacturing, being free of backlash and friction, ultrahigh repeatable precision, compact structure and needless lubrication [9]. As a consequence, we designed the decoupled XY compact parallel micromanipulator (DCPM) with flexure hinges that can transmit movement by resorting to elastic deformation of the mechanism [10]. Moreover, piezoelectric translator (PZT) actuator is the most universal driver that can be used in the precision engineering. The reasons are as below. Firstly, it has high control precision and rapid response. Secondly, it possesses strong driving force, lower rate of work characteristics, etc. However, it has some restrictions in the engineering applications due to the limited distance of travel and hysteresis. In order to resolve this problem, an amplifier is essential for a compliant parallel micromanipulator that will adopt PZT actuators as a driver. The researchers invented many kinds of amplifiers based on different types in order to achieve large displacement, such as a differential mechanism, Scott–Russell mechanism, etc. [11,12,13,14]. However, there are two main approaches to enlarge the output stroke including level principle amplifier [15] and bridge principle amplifier [2], which are usually used in recent years. Although we can get a high magnifying ratio by using a level principle amplifier, but it also loses the linearity of the object when the amplification ratio exceeds a calculated value at the same time [16]. Thus, we adopt the bridge principle amplifier in the structure of the DCPM to satisfy the requirement of being enlarged at a great ratio and it is kept in good linearity.

In addition, the traditional XY manipulator is designed with the serial connection of two different directional stages that would lead to high inertia, low natural frequency and cumulative errors [17]. In addition, there are some papers which concentrate on an XY compliant parallel manipulator [18,19,20]. Based on previous studies, this paper presents a novel parallel architecture to take the place of a serial mechanism in order to overcome such drawbacks. However, the parallel architecture has a coupled motion and a stress stiffening effect, which usually brings nonlinearities to actuation. As we all know, piezoelectric translator (PZT) actuators are easily damaged by the tangential force, and the coupled motion causes the cross-axis error [21]. The method to solve these problems is to design a decoupler to realize the actuation decoupling and the stage motion isolation.

This paper concentrates on the conceptual design of a new high precision decoupled XY compact parallel micromanipulator, which is driven by two PZT actuators. In the remainder of the paper, the mechanical design of DCPM is presented in Section 2. Compliance and stiffness modeling of the flexure mechanism based on the matrix method are performed in Section 3. The comparison of the DCPM performance between the matrix method and finite element analysis (FEA) is given in Section 4. Concluding remarks are presented in Section 5.

## 2. Mechanical Design of the DCPM

### 2.1. Design and Analysis of a Amplifier

**Figure 1:**
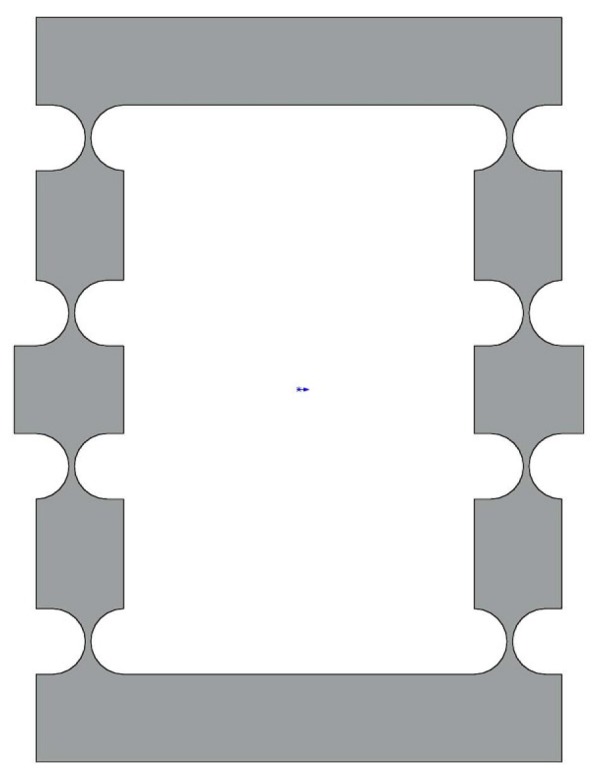
The basic bridge amplifier with right circular hinges.

**Figure 2:**
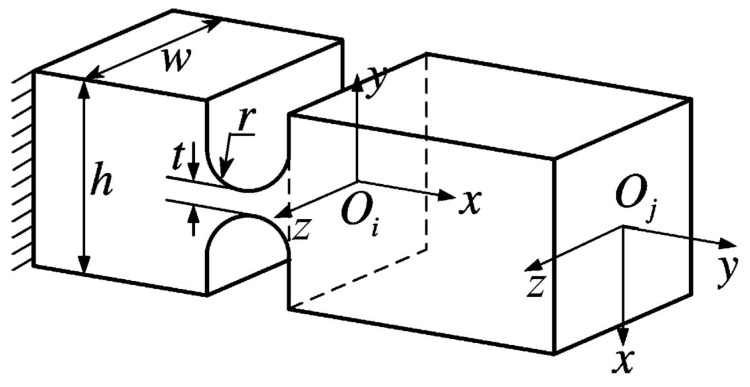
The right circular hinge.

**Figure 3:**
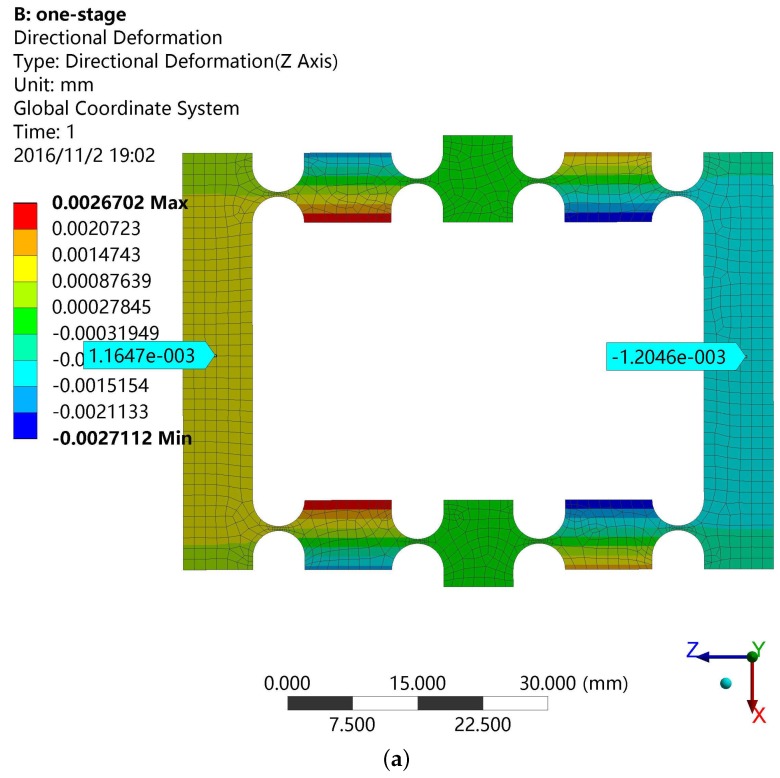

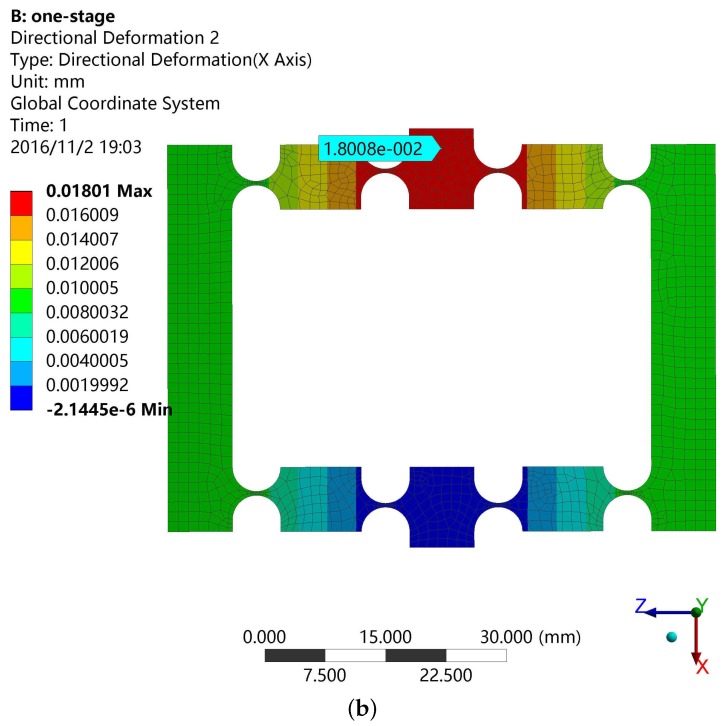
The basic bridge amplifier with right circular hinges. (**a**) The deformation in the *z*-direction; (**b**) the deformation in the *x*-direction.

The challenge that we need to overcome during design of the DCPM is to enlarge the displacement of the end-effector while maintaining a compact size. In order to solve the problem, we adopt the bridge-type amplifier that has been introduced in [22,23], because this kind of amplifier has a number of advantages such as compact size and large amplification ratio. The mechanism to enlarge the displacement is presented in Figure 1, which is designed with the right circular hinge, as shown in Figure 2, due to the higher precision in rotation. The parameters of the basic mechanism are shown in Table 1. We can get the input stiffness and the amplification ratio with FEA simulation, and the result is tabulated in Table 2 and Figure 3.

### 2.2. The Series Connection of the Basic Mechanism

**Figure 4:**
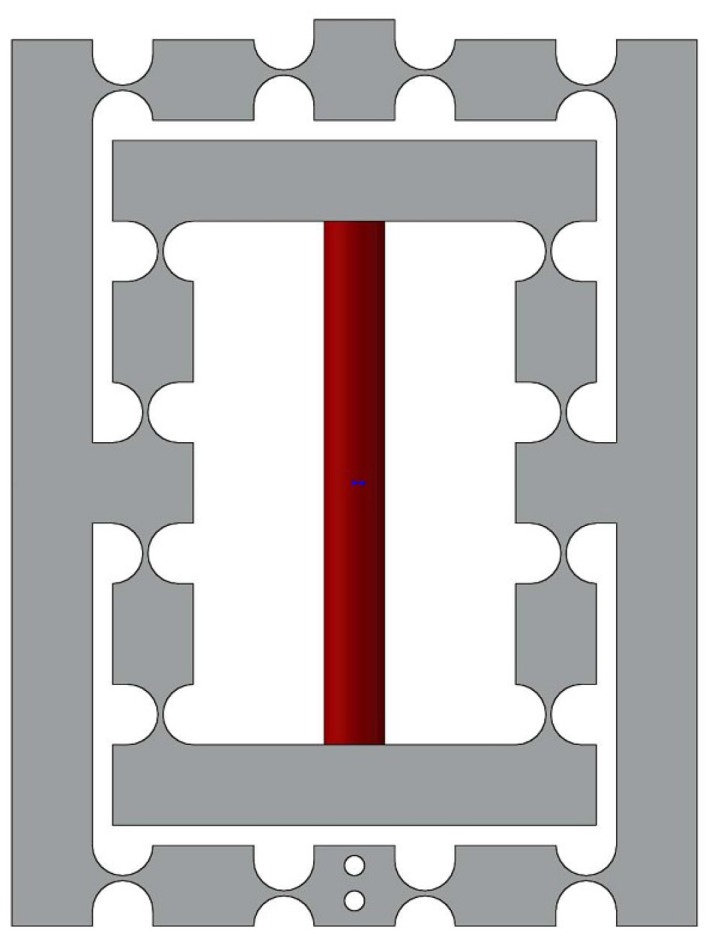
Two-stage bridge type of the amplifier (the red part is piezoelectric translator (PZT) actuator).

**Figure 5:**
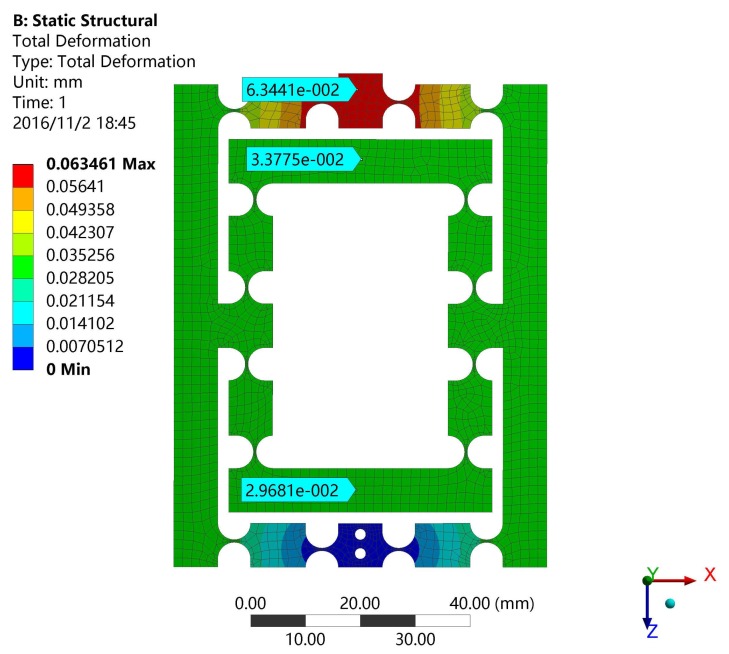
Simulation results of the two-stage amplifier.

The one-stage amplifier does not satisfy our requirements in some applications such as having a lack of stiffness and an insufficient amplification ratio. For this reason, we designed a series connection of the basic mechanism to achieve maximum stroke and maintained the compact size at the meantime. The model of the two-stage amplifier is illustrated in Figure 4. Then, the design requirement is verified by FEA simulation. The simulation results of two types of amplifiers are summarized in Table 3 and Figure 5. The enlarged stroke effect of the two-stage amplifier is obviously better than the one-stage amplifier. As a result, we employed a two-stage amplifier in designing the DCPM.

### 2.3. Design of the Decoupled Flexure Mechanism

**Figure 6:**
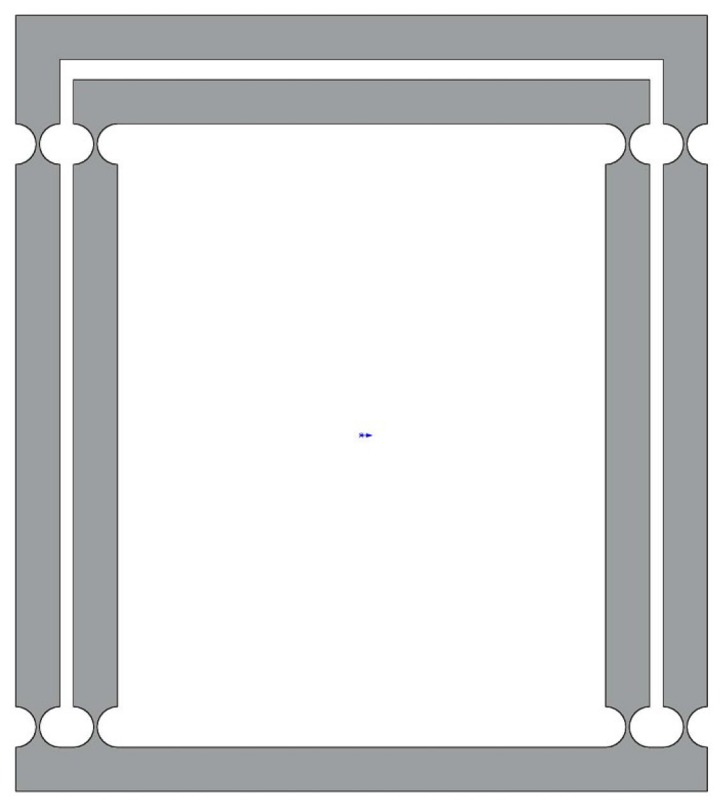
The decoupled flexure mechanism.

In the process of operating a XY stage, the parasitic motion has a negative effect on the operation accuracy. In order to alleviate the transverse loads of the actuator and reduce the parasitic motions, a decoupled mechanism is necessary in the design. Then, a type of decoupler with a right-circle flexure hinge is presented as shown in Figure 6, which is referred to [17]. It has a number of merits including compact size, significant relief of the parasitic motions and transverse loads.

### 2.4. A New High Precision Decoupled XY Compact Parallel Micromanipulator

**Figure 7:**
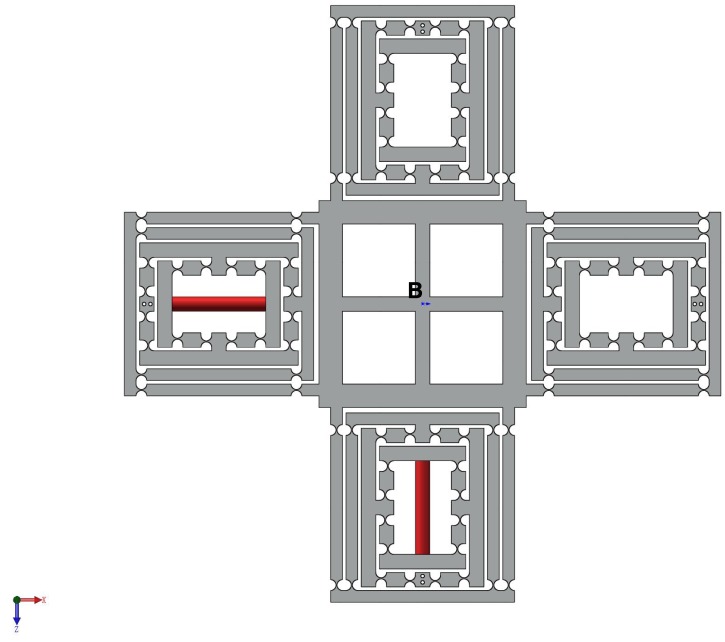
The new high precision decoupled XY compact parallel micromanipulator (the red part is PZT).

A new type of DCPM is proposed as shown in Figure 7, which is comprised of four PZTs, the stroke amplifier, the decouple mechanism and the output platform.

## 3. Compliance and Stiffness Modeling of the Flexure Mechanism Based on the Matrix Method

There are a variety of methods to model the compliant mechanism [24]. For example, the pseudorigid body (PRB) method is a widely used approach that utilizes the simplification principle. However, the PRB method has disadvantages such as it can not perform a complete compliance and stiffness analysis for the reasons that it only concerns compliance in the working directions. At this point, a method is proposed which not only considers the deformation of each component of the stage but also can be derived effectively. In what follows, a lumped model based on the matrix method is demonstrated and verified on the DCPM.

We have learned that a flexure hinge element can be expressed by a 6×6 compliance/stiffness matrix model in its local coordinate system. The compliance matrix has been derived in some previous literature as follows:

(1) $$C_{h}=\left[\begin{array}{cccccc} \frac{\Delta x}{\Delta F_{x}} & 0 & 0 & 0 & 0 & 0\\ 0 & \frac{\Delta y}{\Delta F_{y}} & 0 & 0 & 0 & \frac{\Delta y}{\Delta M_{z}}\\ 0 & 0 & \frac{\Delta z}{\Delta F_{z}} & 0 & \frac{\Delta z}{\Delta M_{y}} & 0\\ 0 & 0 & 0 & \frac{\Delta \theta_{x}}{\Delta M_{x}} & 0 & 0\\ 0 & 0 & \frac{\Delta \theta_{y}}{\Delta F_{z}} & 0 & \frac{\Delta \theta_{y}}{\Delta M_{y}} & 0\\ 0 & \frac{\Delta \theta_{z}}{\Delta F_{y}} & 0 & 0 & 0 & \frac{\Delta \theta_{z}}{\Delta M_{z}} \end{array}\right],$$

where the compliance factors in the matrix are referred to [25]. It is easy to make a diagonal by choosing a proper local coordinate system for the compliance matrix [26]. In particular, the compliance factors $C_{h}\left(1\right.,\left.1\right)$, $C_{h}\left(2\right.,\left.2\right)$, and $C_{h}\left(6\right.,\left.6\right)$, which are the most valuable elements that can construct a diagonal form to reduce the calculating cost.

According to Figure 2, the local compliance with respect to the ground can be written as $C_{i}^{0}=C_{h}$, where the under-right superscript indicates the fix-end, and the upper-right superscript denotes the free-end. In other words, “0” represents the ground that is the fix-end. “*i*” indicates the frame $O_{i}$ that is the free-end. In addition, it is known that the formula that will transform frame $O_{i}$ into frame $O_{j}$ is

(2) $$C_{j}=T_{i}^{j}C_{i}{\left(T_{i}^{j}\right)}^{T},$$

where the transformation matrix $T_{i}^{j}$ can be written as

(3) $$T_{i}^{j}=\left[\begin{array}{cc} R_{i}^{j} & S\left(r_{i}^{j}\right)R_{i}^{j}\\ 0 & R_{i}^{j} \end{array}\right],$$

where $R_{i}^{j}$ is the rotational matrix of frame $O_{i}$ relative to frame $O_{j}$, and $S\left(r\right)$ indicates that the skew-symmetric operator takes on the following form:

(4) $$S\left(r\right)=\left[\begin{array}{ccc} 0 & -r_{z} & r_{y}\\ r_{z} & 0 & -r_{x}\\ -r_{y} & r_{x} & 0 \end{array}\right].$$

Hence, the formula to calculate the displacement $\mu_{i}$ when applying the external force $F_{0}$ on point 0 is derived by

(5) $$\mu_{i}=C_{i}F_{0}.$$

From the above, the output compliance $C_{B}$ can be gained by calculating the compliance of point B relative to the ground. On the other hand, the input stiffness $K_{A}$ can be gained by calculating the stiffness of input-end A relative to the ground.

### 3.1. Output Compliance Analysis

#### 3.1.1. Output Compliance of the Amplifier

**Figure 8:**
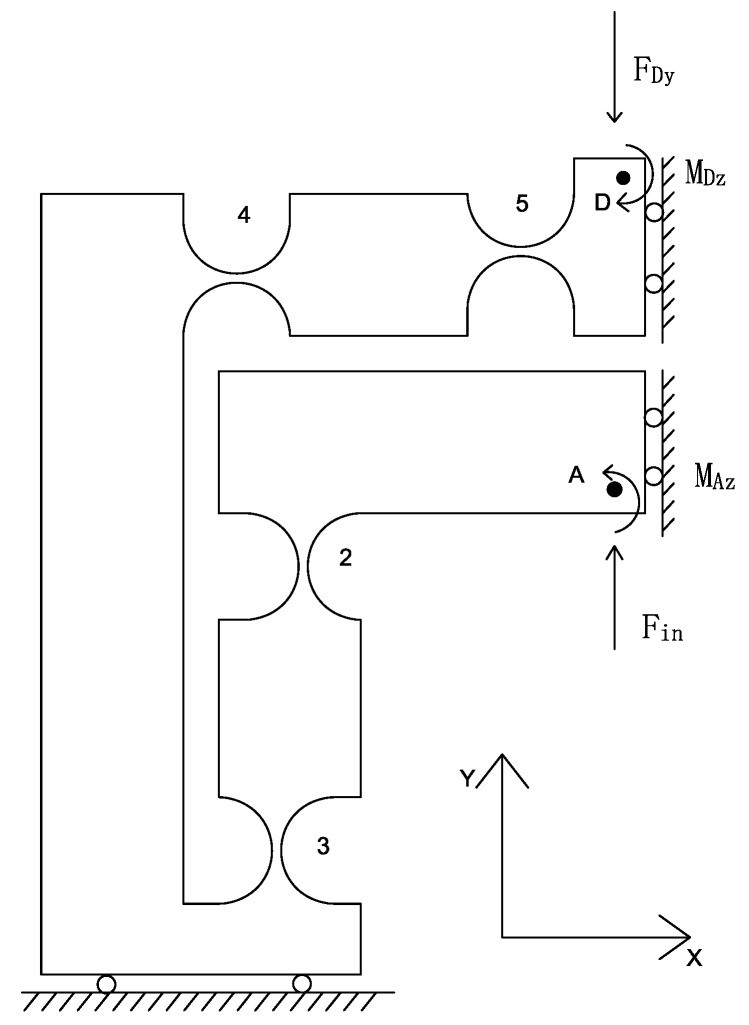
Coordinates for one quarter of the displacement amplifier.

To calculate the output compliance of the amplifier, one quarter of the amplifier is separated and selected (Figure 8), due to the structure of the mechanism is symmetrical. As shown in Figure 8, owing to hinges 2, 3, 4 and 5 are serial connections, the compliance of point D with respect to point A can be derived as

(6) $$\begin{array}{c} C_{D}^{A}=T_{5}^{D}C_{5}{\left(T_{5}^{D}\right)}^{T}+T_{4}^{D}C_{4}{\left(T_{4}^{D}\right)}^{T}+T_{3}^{D}C_{3}{\left(T_{3}^{D}\right)}^{T}+T_{2}^{D}C_{2}{\left(T_{2}^{D}\right)}^{T}, \end{array}$$

where $T_{i}^{D}$ is the transformation matrix and the $C_{i}=C_{h}$$\left(i=2,3,4,5\right)$.

Assume that ${}_{l}C_{D}$ is the output compliance of the left part of mechanism. Then, we can obtain the compliance of the ${}_{l}C_{D}$ due to the structural symmetry:

(7) $${}_{l}C_{D}=C_{D}^{A}+T_{d}^{t}\left(C_{D}^{A}\right){\left(T_{d}^{t}\right)}^{T},$$

where $T_{d}^{t}$ is the transformation matrix that can transform the top part to the down part:

(8) $$T_{d}^{t}=\left[\begin{array}{cc} R_{x}\left(\pi \right) & 0\\ 0 & R_{x}\left(\pi \right) \end{array}\right].$$

Then, due to the parallel connection of the left-right sections of the amplifier, the compliance of the amplifier can be written as

(9) $$C_{D}={\left[{\left({}_{l}C_{D}\right)}^{-1}+{\left(T_{r}^{l}\right)}^{-T}{\left({}_{l}C_{D}\right)}^{-1}{\left(T_{r}^{l}\right)}^{-1}\right]}^{-1},$$

where $T_{r}^{l}$ is the transformation matrix from the right to left part, which can be generated as

(10) $$T_{r}^{l}=\left[\begin{array}{cc} R_{y}\left(\pi \right) & 0\\ 0 & R_{y}\left(\pi \right) \end{array}\right].$$

#### 3.1.2. Output Compliance of the XY Stage

As shown in Figure 7, it is convenient to obtain the compliance of the XY stage, considering one individual limb connected at point B. In addition, the compliance $C_{B}$ can be derived by taking into account that four limbs are connected to the primary stage in the reference coordinate system B, respectively.

### 3.2. Input Stiffness Analysis

The way to analyze the input stiffness of the XY stage is similar to the output compliance modeling. Firstly, the compliance of the whole stage excluding one of the amplifier can be written as

(11) $$C_{D}=C_{D}^{B}+T_{B}^{D}{\left(K_{1B}\right)}^{-1}{\left(T_{B}^{D}\right)}^{T},$$

where $K_{1B}$ is the stiffness of the stage except limb 2, it is supposed that the point D is fixed. As shown in Figure 8, we can obtain the kinematic equations:

(12) $$\mu_{in}=c_{11}F_{in}+c_{12}F_{Dy}+c_{16}M_{Az},$$

(13) $$\mu_{Ay}=c_{21}F_{in}+c_{22}F_{Dy}+c_{26}M_{Az},$$

(14) $$\theta_{Az}=c_{61}F_{in}+c_{62}F_{Dy}+c_{66}M_{Az}=0,$$

which $c_{ij}$ (i,j=1,2,6) is the *i*th row and *j*th column factors of the matrix $C_{A}^{D}$ .

Combining Equations (12)–(14) with Equation (15):

(15) $$\mu_{Ay}=-d_{22}F_{Dy},$$

where $d_{22}=C_{D}\left(2,2\right)$, and the value of the variable $\mu_{in},\mu_{Ay},F_{Dy},M_{Az}$ can be obtained by solving the equation set. Then, the input compliance of the XY stage $C_{in}$ is derived as

(16) $$\begin{array}{c} C_{in}=\frac{\mu_{in}}{F_{in}}=c_{11}-\frac{c_{16}c_{61}}{c_{66}}-\left(c_{12}-\frac{c_{16}c_{62}}{c_{66}}\right)\frac{c_{21}c_{66}-c_{26}c_{61}}{c_{22}c_{66}-c_{26}c_{62}+d_{22}c_{66}} \end{array}$$

so the input stiffness $K_{in}=\frac{1}{C_{in}}$ can be obtained.

Therefore, the displacement $\mu_{Ay}$ can be derived by the input displacement $\mu_{in}$ as

(17) $$\mu_{Ay}=A_{a}\mu_{in}=\frac{d_{22}\left(c_{21}c_{66}-c_{26}c_{61}\right)\mu_{in}}{\left(c_{22}c_{66}-c_{26}c_{62}+d_{22}c_{66}\right)C_{in}},$$

where $A_{a}$ indicates the amplification ratio of the amplifier.

### 3.3. The Amplification Ratio of the XY Stage

In view of the equations written above, the output stroke of point B can be derived as:

(18) $$\mu_{By}=\frac{b_{22}}{d_{22}}\mu_{Dy},$$

in which $b_{22}$ is the factor of compliance matrix ${}_{L1}C_{B}$. As a consequence, taking into account Equations (17) and (18), as well as considering $\mu_{Dy}=\mu_{Ay}$ , the ratio of the enlarged stroke for the stage can be obtained as

(19) $$A_{s}=\frac{\mu_{By}}{\mu_{in}}=\frac{b_{22}\left(c_{21}c_{66}-c_{26}c_{61}\right)}{\left(c_{22}c_{66}-c_{26}c_{62}+d_{22}c_{66}\right)C_{in}}.$$

## 4. Finite Element Analysis (FEA) Validation

**Figure 9:**
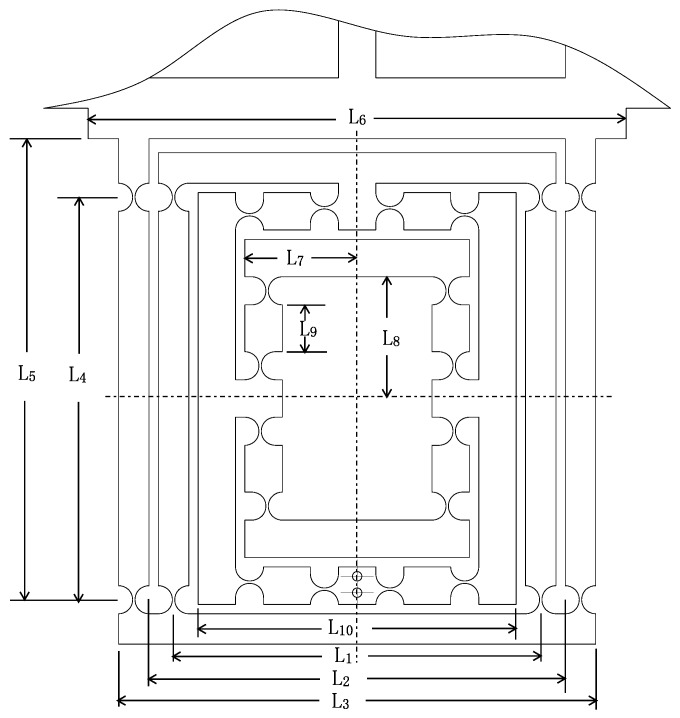
The main structural parameters of an XY decoupled compact parallel micromanipulator (DCPM).

In this section, the finite element analysis (FEA) is used to validate the models of output compliance, input stiffness and the amplification ratio of DCPM. The software adopted in the paper is ANSYS (13.0, ANSYS Inc., Canonsburg, PA, USA), which is one of the most popular finite element analysis software programs being used. The main structure and material parameters of DCPM are given in the Table 4 and Figure 9, where the hinges have adopted the classical one as shown in Table 1.

**Figure 10:**
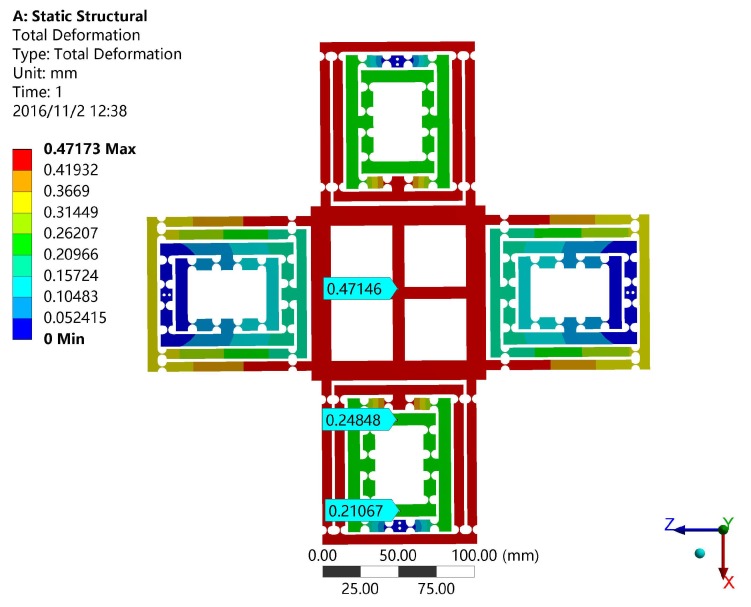
The output compliance simulation results of DCPM.

**Figure 11:**
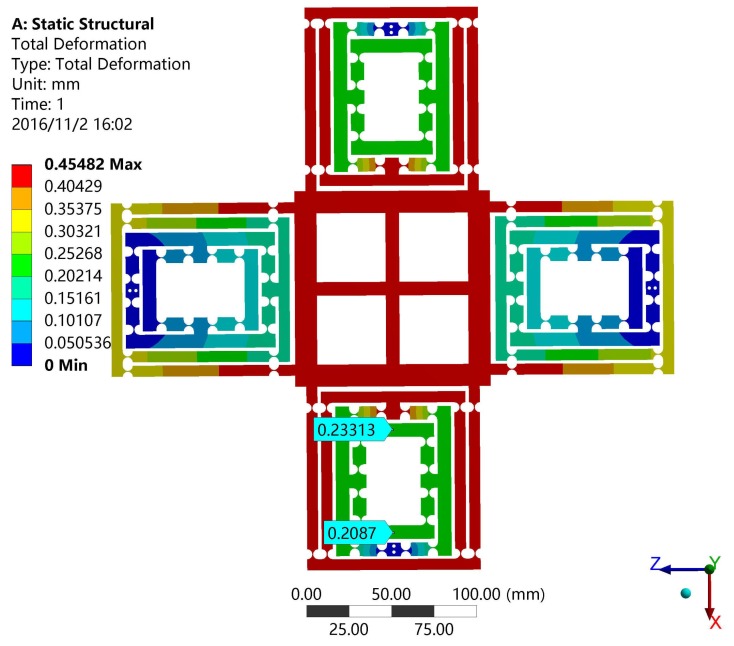
The input stiffness simulation results of DCPM.

For instance, the input load and the output displacement can be obtained by applying a certain distance on the input faces, which determines the output compliance and amplification ratio of the XY stage as shown in Figure 10. It is obvious that the input distance is 0.03781 mm and the output distance is 0.47146 mm. In the same way, the input stiffness of the stage can be validated by FEA as shown in Figure 11. The input distance is 0.02443 mm.

A comparison of the performance between the matrix model method and the FEA method is described in Table 5. The FEA results are defined as the benchmark, and we can improve the design approach according to the errors between the theoretical values and the simulation values.

## 5. Discussion

This paper adopts comparative experiments to analyze the amplification ratio of two kinds of amplifiers. Then, we get the result that the two-stage amplifier is better than the one-stage amplifier. Finally, the FEA results of the DCPM demonstrate that the compliance and stiffness are approximated to the theoretical calculating values. The lumped-compliance mechanism has some advantages such as increasing the precision of kinematics, since distributed compliance has larger unwanted motion and reduces the structure space because the lumped compliance mechanism has more compact structure that utilizes the space efficiently. As for the question about whether the interspace of the lumped structure can satisfy the large motion requirement, the conclusion is gained from the results of simulations that reveal that interference phenomenon does not exist in the experiments. The original contribution of the paper is that we design a novel decoupled XY compact parallel micromanipulator that has many merits including compact structure, certain output compliances, input stiffness and a huge amplification ratio compared with the similar stage in [13,14,17,27]. Compared with the 2-degrees of freedom (DOF) compliant parallel micromanipulator in [13], the similarity is that two stages both have decoupled mechanisms and the difference is that the stage presented in this paper has the two-stage amplifier. The manipulator presented in [14] is also analyzed with the matrix method, but it has a different decoupled mechanism and does not have amplifier. The stage proposed in [17] only has a one-stage amplifier that has a limited amplification ratio compared with our micromanipulator. The similarity is that the two papers both adopted the matrix method to analyze the stage characteristics. The manipulator presented in [27] used the lever mechanism, which is different from the micromanipulator proposed in this paper that adopted the bridge amplifier to enlarge the stroke. The similarity is that they both have two-stage amplifiers.

## 6. Conclusions

This paper presents a novel design of a type of decoupled XY compact parallel micromanipulator (DCPM). The design procedure of DCPM is in favor of optimizing the dimension of the XY stage by calculating input stiffness and output compliance based on the matrix method. Afterwards, the result of matrix method is validated by FEA. The results of the designed XY stage are better than the previous work as the amplification ratio of 12.5 is larger than the value in [27].

In the future, further works will concentrate on such aspects as given below:

(1) Optimizing the parameters of the XY stage to obtain better performance;
(2) Conducting analytical methodology for dynamics analysis;
(3) Experiments will be carried out for a prototype fabrication;
(4) A control system will be designed and conducted by applying different control methods.

## Figures and Tables

**Table 1:** Parameters of the basic bridge amplifier (mm).

*r*	*t*	*h*	*w*
3	0.5	8	12.7

**Table 2:** Simulation results of the basic one-stage amplifier.

Performance	Input Stiffness (N/μm)	Amplification Ratio
Finite-Element Analysis (FEA)	4.22	7.5

**Table 3:** Simulation results of two kinds of amplifiers.

Performance	Input Stiffness (N/μm)	Amplification Ratio
one-stage amplifier	4.22	7.5
two-stage amplifier	9.77	15.5

**Table 4:** The main structure and material parameters of an XY decoupled compact parallel micromanipulator (DCPM).

**Structure Parameters (mm)**				
$L_{1}$	$L_{2}$	$L_{3}$	$L_{4}$	$L_{5}$
78.5	89	102	86	98.5
$L_{6}$	$L_{7}$	$L_{8}$	$L_{9}$	$L_{10}$
115	24	26	10	68
**Material Parameters**				
**Young’s Modulus**	**Yield Strength**	**Poisson’s Ratio**	**Density**	
71.7 GPa	503 MPa	0.33	2810 Kg/m${}^{3}$	

**Table 5:** The performance comparison of the proposed DCPM.

Items	Output Compliance $C_{B}$ (μ/N)	Input Stiffness (N/μm)	Amplification Ratio
Matrix model	1.02	12.7	13.5
FEA	0.94	13.22	12.5
Deviation$\left(\%\right)$	7.8	4.1	7.4
